# Integrative Effect of Protective Structures and Irrigation Levels on Tomato Performance in Indian Hot-Arid Region

**DOI:** 10.3390/plants11202743

**Published:** 2022-10-17

**Authors:** Pratapsingh S. Khapte, Pradeep Kumar, Akath Singh, Goraksha C. Wakchaure, Anurag Saxena, Leo Sabatino

**Affiliations:** 1 ICAR—National Institute of Abiotic Stress Management, Baramati 413115, India; 2 ICAR—Central Arid Zone Research Institute, Jodhpur 342003, India; 3 ICAR—National Dairy Research Institute, Karnal 132001, India; 4 Department of Agricultural, Food and Forest Sciences, University of Palermo, 90128 Palermo, Italy

**Keywords:** greenhouse cultivation, growth, fruit quality, net house, deficit irrigation

## Abstract

Protected cultivation is gaining momentum in (semi) arid regions to ameliorate the adverse environmental impacts on vegetable crops, besides ensuring high resource use efficiency in resource-limiting environments. Among the less techno-intensive protected cultivation structures, naturally ventilated polyhouses (NVP), insect-proof net houses (IPN) and shade net houses (SNH) are commercial structures in India. With the aim to find the best-protected structure, together with optimum irrigation level, for high yield and water productivity of the tomato crop, the most popular crop in hot arid regions, we evaluated tomato performance in low-tech protected structures (NVP, IPN and SNH) in interaction with three irrigation levels (100, 80 and 60% of crop evapotranspiration, ETc) during spring–summer of 2019 and 2020. The NVP was found superior to both the net house structures (IPN and SNH) for different performance indicators of tomatoes under investigation. The components of plant growth (leaf and stem dry mass) and fruit yield (fruit size, weight, yield), as well as fruit quality (total soluble solids, fruit dry matter and lycopene content) were higher in NVP, regardless of irrigation level. The yield as well as water productivity were significantly higher in NVP at 100% ETc. However, there was no statistical variation for water productivity between NVP and IPN. Microclimate parameters (temperature, relative humidity and photosynthetic active radiation) were markedly more congenial for tomato cultivation in NVP followed by IPN in relation to SNH. Consequently, plants’ physiological functioning with higher leaf relative water content (RWC) and lower leaf water potential concomitantly with better photosynthetic efficiency (chlorophyll fluorescence, F_v_/F_m_), was in NVP and IPN. Most growth and yield attributes were depressed with the decrease in water application rates; hence, deficit irrigation in these low-tech protected structures is not feasible. For tomato cultivation in resource-scarce arid regions, the combination of the normal rate of irrigation (100% ETc) and NVP was optimal for gaining high yield as well as water productivity as compared to net houses.

## 1. Introduction

The protected cultivation of vegetable crops is increasing at a fast rate around the globe, more particularly in the harsh and non-congenial environments of arid and semi-arid regions, where open field cultivation is constrained by various external crop-limiting factors. Considering the nutritional importance of the tomato, as well as its economic security, it is widespread in protected cultivation. The majority of arid and semi-arid regions of the world are increasingly becoming scarce in natural resources, mainly water [1]. High temperature, intense solar radiation, low precipitation and relative low humidity are the key characteristic features of an arid ecosystem that render the growth and development of vegetable crops unsuitable [2]. Nevertheless, as the global population increases, these regions need to meet the escalating food demand. Expanding the cultivation of vegetables adopting protected cultivation systems and therefore reducing the adverse effect of climatic conditions can be a key factor to accomplish an efficient use of dwindling land and water resources.

Different types of protected cultivation structures are currently used, ranging from climate-controlled greenhouses with sophisticated equipment to plastic/net-covered low-tech structures including rain shelters/tunnels [3]. Climate controlled greenhouses, including semi-hi-tech fan-and-pad greenhouses, are less common protected cultivation structures for commercial vegetable production in Indian (semi) arid regions due to their high water, energy and skills requirements [2,3]. Except for a few large organized greenhouse setups run by the corporates, the greenhouse industry in India is mostly an unorganized sector, and individual farmers mostly operate on an average one-to-two-acre-sized protected cultivation structure. Low techno- and energy-intensive protected structures such as naturally ventilated plastic greenhouses (polyhouse) and insect net and shade net houses of different sizes are common and feasible protected cultivation structures for most of the farmers in India [3].

In low-tech protected structures, climatic alterations are often modulated by the covering (cladding) material. In this respect, crop growth and development and yield are highly affected by the growing environment [4,5]. The cladding material of the structures is classified based on its purpose and properties. The naturally ventilated polyhouse (NVP) is covered with polyethylene film and is provided with a layer of insect-proof net on its side-walls for ventilation. This facilitates alterations in the micro-climatic variables, primarily diffuse radiation and relative humidity, whereas net houses such as insect-proof net houses (IPN) or shade net houses (SNH) usually cut direct solar radiation and manipulate light intensity and quality with the least control on humidity [3]. However, microclimate modification inside these low-tech protected structures can differ, as they are often influenced by diverse outside environments [3,4]. Accordingly, in a study conducted during post monsoon—autumn season (August–November) in the Indian arid region, the measured microclimate variables, i.e., temperature, relative humidity and photosynthetic active radiation at different phenological stages were relatively in a more favorable range in NVP as compared to IPN and SNH. Furthermore, gynoecious cucumber yields in NVP were 42 and 142% higher as compared to those in IPN and SNH [4]. Thus, it is agreeable that under a specific climate, different protected structures should control microclimatic variables including photosynthetic active radiation, temperature, relative humidity and their interaction across the day and cropping season. Therefore, it should be emphasized that the protected structure must be optimized based on the local climatic conditions for vegetable crops such as the tomato [6,7].

The main purpose of protected cultivation systems has been growing crops beyond their normal season or extending the growing season so as to ensure year-round supply of vegetables, hence overall profitability [1,8]. Tomatoes, both normal and cherry, mini-cucumber and capsicum, are the most popular greenhouse vegetables in India, as with most other parts of the world. These crops are mostly grown on soil-beds but some modern greenhouses adopt coir or cocopeat-based soilless culture. In most parts of the northern Indian plains, particularly (semi) arid regions, open field tomato cultivation in spring–summer is very challenging owing to high day temperatures (>40 °C) coupled with hot winds. Hence, tomato growing under protected conditions becomes inevitable [1]. The optimum temperature range for tomato production is 25–30 °C/20 °C (day/night) and >35 °C is detrimental to growth and development [9,10]. Flowering, fertilization and growth of fertilized embryos in tomatoes are hampered beyond the upper temperature limit as flowering and fruit set stages are highly sensitive to high temperatures [9]. Furthermore, reproductive failure under high-temperature stress is widely associated with morpho-anatomical changes and fruit yield reduction. Hence, under such a harsh arid and semi-arid environment, protected structures with suitable coverings (with glass or polycarbonate sheeting, the commonly used plastic or net, or the combination plastic and shade net) can positively modulate microclimate variables, thus favorably affecting the plant growth and development process and finally the yield.

Water is the most important input for vegetable production in resource-constrained arid and semi-arid regions. In contrast to semi-arid regions, arid regions have significantly higher evapotranspiration rates and low precipitation; therefore, increasing the efficiency of water use for crop production is vital [11]. Nevertheless, it is well recognized that the efficiency of water use in protected cultivation structures is very high, although it is influenced by several factors including region, design of protected structure, soil-type, crop species and growing/irrigation practices [12]. For instance, the average water requirement to produce one kilogram of tomatoes under different types of protected environments varies in different countries. In Spain, it is about 40 litres in unheated plastic houses and 27 litres in regulated ventilated unheated plastic houses; in Israel, it is 30 litres in unheated glasshouses, while it is lowest in the Netherlands, requiring 22 litres in climate-controlled glasshouses with CO_2_ enrichment, and 15 litres when it is combined with a re-circulating system [12]. Furthermore, deficit irrigation has been an effective management strategy to economize water use in irrigated agriculture, especially in water-scarce regions. It has been highlighted that tomato production in water-stressed areas is at risk in those situations where deficit irrigation scheduling is necessary to achieve optimal yields [13]. Various reports suggest considerable water savings, with or without witnessing any yield penalties such as in field-grown processing tomatoes [14,15]. In processing tomatoes, deficit irrigation is considered to be a useful tool in order to improve some fruit quality parameters, and a slight reduction in fruit yield is acceptable [14,15].

In contrast, yield maximization per unit of applied resources (e.g., water) has been the primary aim of greenhouse cultivation. However, there are research efforts to determine whether a deficit irrigation strategy would work differently under different types of protected cultivation structures and provide any advantage under different types of protected environments, particularly in terms of water saving and/or fruit quality improvement in low-water-availability zones such as hot-arid regions of India. Remarkably, the interactive effect of different protective structures and the application of various irrigation levels on tomato performance have not yet been examined. Specific combinations of protective structures and irrigation levels might have a positive effect on plant growth, yield and fruit quality. Furthermore, there could be an optimal irrigation level inferior to the standard irrigation level that would allow an improvement in overall plant fitness. Accordingly, we planned this study to assess the effectiveness of three low-tech protected structures coupled with three irrigation levels on tomato production and quality, as well as water productivity during spring–summer in an arid region. The performance was determined based on measured microclimate variables. The treatment effects were also assessed on plant growth and physiological processes and consequently on fruit yield and quality attributes.

## 2. Materials and Methods

### 2.1. Growth Conditions

The research experiments were conducted in three similar size (128 m^2^) low-tech protected cultivation structures, i.e., naturally ventilated polyhouse (plastic greenhouse) (NVP), insect-proof net house (INH) and shade net house (SNH) during spring–summer (January—May) in two years (2019 and 2020) at ICAR-Central Arid Zone Research Institute, Jodhpur (26°15′ N latitude, 72°59′ E longitude). The details of the structures are described in Khapte et al. [3]. The climate of the study area is hot and arid with peculiarity of high diurnal and seasonal temperature variations, less humidity, high wind speed and a high rate of evapotranspiration [1,16]. The microclimatic variables recorded in these structures during the cropping period are presented in Table 1. The air temperature and relative humidity were recorded with an Assmann psychrometer (Model MR-58, Hisamatsu, Tokyo, Japan) and photosynthetically active radiation (PAR) was recorded with a line quantum sensor (MQ-301, Series#1178, Apogee, Logan, UT, USA). The commercially cultivated indeterminate F_1_ tomato hybrid NS-4266 (Namdhari seeds, Bengaluru, India) seeds were sown in 52 mm cell plug-trays filled with soilless medium (vermiculite: cocopeat; 1:2 ratio *v*/*v*). The seedlings were fertigated twice a week with 0.2% water-soluble complex fertilizer N:P:K-19:19:19 (Gromor, Coromandel International Ltd., Secunderabad, India) along with 0.1% micronutrient mixture (Multiplex Group of Companies, Bengaluru, India).

The soil of the experimental site had organic carbon 0.22%, pH 7.8, total N, available P and K 0.03%, 16.3 and 221.5 kg ha^−1^, respectively. The soil contained sand, silt and clay 85%, 8.1% and 5.5%, respectively. According to US soil taxonomy, the soil is classified as coarse-loamy, mixed, hyperthermic Camborthids. At the time of land preparation, 25 t ha^−1^ compost + 1.0 t ha^−1^ neem cake was mixed in the topsoil. The commercial grade water soluble fertilizers 200:200:350 kg ha^−1^, N:P:K along with 50 kg Ca and 30 kg Mg were applied through fertigation during the cropping period. Thirty-day-old seedlings were transplanted in soil on 9” height raised beds in all the structures in a paired row at 50 cm × 50 cm spacing (3.0 plants m^−2^). The single main leader stem was maintained by regular removal of side shoots and supported by twining with plastic thread attached to overhead trellis wire. The crop management operations, including pruning and training, crop protection, etc., were uniformly followed in all the structures.

### 2.2. Plant Growth and Fruit Yield

Five plants were arbitrarily selected and tagged in each replication of a total four replications for the measurement of plant growth and yield related parameters. The experiment was terminated 142 days and 150 days after transplanting in 2019 and 2020, respectively. At the termination, the leaves and stem were separated. The plant parts were dried in an oven at 65 °C until constant weight was obtained, and their dry weight (DW) was determined. Leaf area was recorded using a leaf area meter (LI-3100C, LI-COR, Inc., Lincoln, NE, USA). At each harvest, fruit number and weight were recorded from each tagged plant across all the treatments separately, and by summing them up across all the harvests per plant, fruit number and yield were obtained. Mean fruit weight was computed by dividing total fruit weight by fruit number.

### 2.3. Fruit Quality Parameters

The samples of fifteen arbitrarily selected uniform ripe fruits from each replication were taken from the 4th—5th fruit clusters (105 days after transplanting) to measure the fruits’ physical parameters: fruit longitudinal and transverse diameter (cm), fruit firmness (kg cm^−2^), total soluble solids (TSS, in °Brix) and fruit dry matter (DM, %). Fruit TSS content was determined with a digital handheld refractometer (Bellingham and Stanley, Tunbridge Wells, UK). A drop of fruit juice was placed on a digital refractometer and brix value was noted at room temperature. Fruit firmness was gauged by using a digital fruit hardness tester (model no FR-5120, Lutron Electronic Enterprise Co., Ltd., Taipei, Taiwan). The fruits were punctured with a plunger (6 mm) at two places opposite to each other in the radial axis and the pressure required to puncture was recorded (kg cm^−2^). Fruit DM content was determined by weighing the dried fruit kept in a forced air oven at 70 °C until constant weight was obtained.

The lycopene and total carotenoid contents of fresh tomato fruits were analyzed as per the method described by Ranganna [17]. Briefly, 5 g of tomato fruit pulp of freshly harvested tomato fruits was extracted repeatedly with acetone and transferred to a separating funnel consisting of 20 mL of petroleum ether, mixed gently and two phases were separated. The petroleum extract was transferred to 10 g of anhydrous sodium sulphate and the extract decanted to make up to known volume. Further supernatant was used by reading absorbance at 470 and 503 nm using a UV-vis spectrophotometer, and the values of lycopene and carotenoid contents were expressed in mg 100 g^−1^ FW. Fruit color analysis was performed with a handheld colorimeter (WR10, Shenzhen Wave Optoelectronics Technology Co., Ltd., Shenzhen, China). L* value (black to white), a* value (redness to greenness) and b* value (yellowness to blueness) of the tomato fruit samples were recorded [18].

### 2.4. Physiological Parameters

The physiological status of the plants was recorded at the active growth period (95 days after transplanting). Leaf relative water content (RWC) was determined as per the method described by Khare et al. [19]. Twelve leaf discs for each measurement were weighed to determine the fresh weight (FM) and these were rehydrated in distilled water for 6 h. Then, turgid leaf discs were surface-dried over tissue paper and weighed again to obtain turgid weight (TM). Further, the same discs were oven-dried at 80 °C for a period of 24 h to record dry weight (DM). The determination of total chlorophyll content was made at active growing leaves (4th fully open from the top). Total chlorophyll concentration in fresh leaf tissues was determined following a method suggested by Arnon [20]. Pigment was extracted using 80% aqueous acetone, and estimated by measuring absorbance at 645 nm and 663 nm on spectrophotometer and concentration was expressed in µg mL^−1^ fresh weight.

During the active growth period of the crop at reproductive stage, F_v_/F_m_ was measured with a chlorophyll fluorescence meter (OS-30p, Opti-Sciences, Inc., Hudson, NH, USA). The leaf water potential was measured with a pressure chamber (Model 600, PMS Instrument Co. Corvallis, OR, USA).

### 2.5. Irrigation Treatment and Water Productivity

Irrigation water was applied through inline drip laterals with an emitter capacity of 1 L per hour, with 30 cm spacing between the emitter, every day in the morning hours (Table 2). The reference pan from the meteorological observatory located about 100 m away from the protected structures was used to estimate standard crop evapotranspiration (i.e., 100% ETc), and accordingly the amount of irrigation water for irrigation levels 100, 80 and 60% of ETc, which were uniformly applied in all the protected structures, was calculated.

Water productivity (WP, kg m^−3^) was computed according to Dermitas and Ayas [21], and was expressed as a ratio of total yields to total water applied, including irrigation water, during the entire growing period.

### 2.6. Statistical Analysis

The research data of the experiment were statistically analyzed by analysis of variance using SPSS software package (SPSS Ver. 20). Duncan’s Multiple Range Test was performed and mean values of three replicates within columns were separated (*p* ≤ 0.05). The descriptive statistics and homogeneity tests were applied while analyzing the data.

## 3. Results

### 3.1. Microclimate Parameters

The microclimatic variables inside the protected environment structures were significantly affected by the cladding materials (Table 1). The mean maximum and minimum temperatures inside the structures gradually increased with the transition of seasons from winter to summer in all the structures. The mean relative humidity (RH) was highest in the month of January and thereafter decreased up to the end of cropping season. The photosynthetically active radiation (PAR) was highest in the months of February and March in NVP and IPN, whereas it later decreased from April because there was provision of mobile shade net inside these structures. However, in SNH it was continuously higher until May in both the years.

### 3.2. Plant Growth Parameters

**Figure 1:**
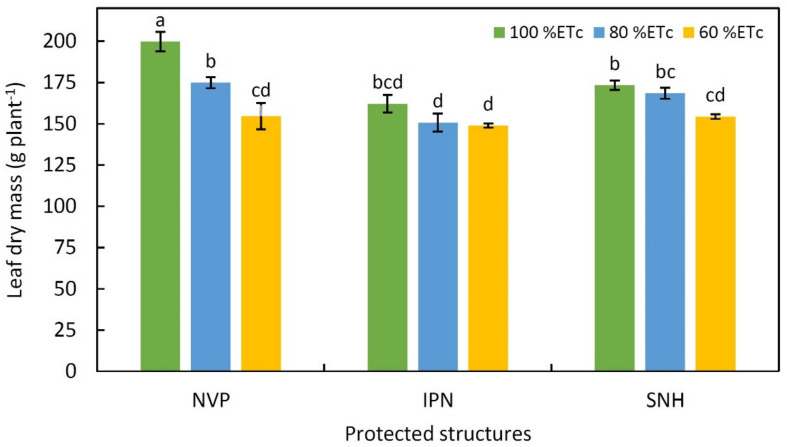
Leaf dry mass (g plant^−1^) under different irrigation levels and protected environment structures. The values followed by the same letter are not significantly different at *p* ≤ 0.05 according to DMRT. Protected structures: NVP, Naturally ventilated polyhouse; IPN, Insect-proof net house; and SNH, Shade net house.

The obtained results for stem dry mass (DM), leaf area, plant length, node number and stem girth of tomato in response to different structures and irrigation levels are summarized in Table 3 and Table 4. In both the years, the interactive effect of structures and irrigation levels on most plant growth parameters was not significant, except for leaf and stem DM, though showed inconsistent effects with respect to individual years and the two pooled years (Table 3, Figure 1). The comparative values of growth parameters were slightly higher in the year 2020 than 2019 for most of the growth parameters with no significant variation between the years. Stem DM was significantly higher in NVP than IPN and SNH, while leaf area was distinctly higher in NVP and SNH than IPN in both years as well as in pooled data. In the two-year pooled data, stem DM and leaf area significantly decreased with respect to decrease in water supply, but the response was inconsistent in the two years between deficit irrigation levels (80 and 60% ETc) (Table 3). No significant effect of the protected structure was noticed on plant height, while the response to irrigation level was significantly lower plant height observed at 60% ETc (Table 4). Further, both the node number and stem girth were significantly affected by structure and irrigation levels, but differently. Node number was distinctly higher in NVP than IPN or SNH in both years as well as in their pooled data. While, compared with 100% ETc irrigation level, stem girth was significantly reduced only under 60% ETc in both years and in their pooled data (Table 4).

### 3.3. Fruit Physical Parameters

The fruit physical parameters for two years and their pooled data are shown in Table 5. It is very clear that tomato fruits from the NVP displayed significantly higher fruit physical parameters such as fruit longitudinal diameter (LD), transverse diameter (TD) and maximal fruit firmness as per the two-year pooled data. Both LD and TD values were distinctly higher in NVP than IPN and then SNH, whereas fruit firmness was only significantly higher in NVP than IPN or SHN in the pooled and in 2019 data. Based on two-year pooled data analysis, it is clear that with decreasing water supply from 100% ETc to 80 and 60% ETc, fruit LD and TD will decrease, while fruit firmness increases with water deficit only at 60% ETc in both years, as well as their pooled data (Table 5).

### 3.4. Fruit Quality Parameters

Among the three different protected structures and irrigation levels, fruit quality parameters differed significantly (Table 6). As per the pooled data, the fruit dry DM, total soluble solids (TSS) and lycopene contents were significantly higher in NVP; however, the total carotenoids showed an opposite trend with distinctly higher content recorded in NVP followed by IPN and SNH, but no significant variation in carotene content between IPN and SNH was observed in either of the years (Table 6). Further, the two-year pooled data (Table 6) indicates that there was an improvement in some fruit quality parameters such as fruit DM, TSS and total carotenoid contents under deficit irrigation. The fruit DM and TSS contents were significantly increased only at 60% ETc level as compared to 100 and 80% ETc, while total carotenoid content increased in equal quantity at both 80 and 60% ETc. In contrast, the increase in the level of water stresses from 100 to 80 and 60% ETc led to a decrease in lycopene content accumulation in the tomato fruit as evident in the pooled data (Table 6).

The results of fruit color components as affected by protected structures and irrigation levels determined only in cropping season 2020 are reported in Table 7. The assessment of tomato fruit color from the protected structures clearly illustrated that fruit from SNH had distinctly higher L* value and the least a* value, showing contrasting features with those obtained from NVP. Based on CIE-LAB color space, the distinctly higher L* value along with slightly higher b* value of fruits from SNH suggest that fruits were lighter and yellowish in color in contrast to the dark red fruits obtained from NVP. The higher value a* of fruits from the NVP correlates with the lycopene content, where it was maximal, which indicates the redness of the fruits. Therefore, fruit quality was significantly better in NVP, having a uniform red color as compared to the other structures. The fruits from normal irrigation exhibited a higher hue (L*) and lower value of a* whereas fruit from the water-stressed deficit condition were dark red with lower L* and higher a* coordinate values.

The effect of protected cultivation structures was more pronounced than irrigation levels for tomato plant physiology status (Table 8). The leaf relative water content (RWC) and efficiency of photosystem II (PSII, F_v_/F_m_) are the important water stress indicators, and these were highest in NVP and lowest SNH in both years as well as in the pooled data. The leaf water potential (LWP) and total chlorophyll content were measured lowest in NVP in both years as well as in the pooled data. Among irrigation levels, RWC and F_v_/F_m_ were highest while total chlorophyll and LWP were lowest under the normal water supply condition (i.e., 100% ETc).

### 3.5. Fruit Yield Parameters

**Figure 2:**
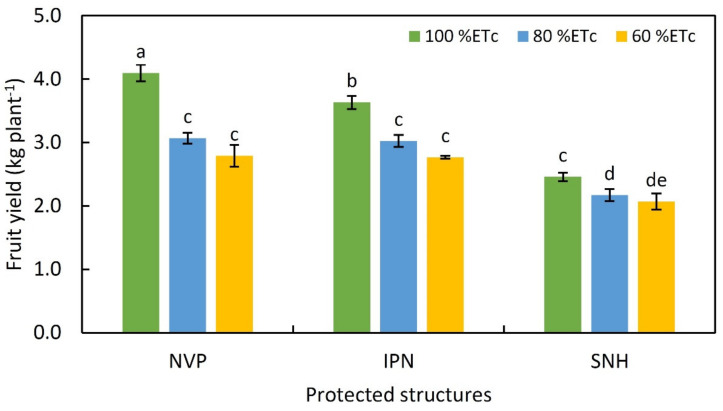
Total fruit yield (kg plant^−1^) in protected environment structures at different levels of irrigation levels. Mean values followed by the same letter are not significantly different at *p* ≤ 0.05 according to DMRT. NVP, Naturally ventilated polyhouse; IPN, Insect-proof net house; and SNH, Shade net house.

The protected structures and irrigation levels significantly affected tomato fruit yield attributes (Table 9). Total fruit yield was significantly affected by the interaction of structure and irrigation level in 2020 and in the pooled data (Table 9; Figure 2), while the number of fruits per plant showed an interaction effect only in the pooled data of the two years (Table 9).

The yield parameters, such as fruit number, mean fruit weight and fruit yield, were higher in the year 2020 than 2019; however, the trend exhibited by them was almost similar with respect to the effects of treatments. Interestingly, the fruit yield per plant was higher in NVP, but statistically similar to IPN, during both years as well as in the pooled data. The increment for fruit number plant^−1^ in NVP and IPN in comparison with SNH was 48% and 41%, respectively. Further, irrigation levels also impacted the fruit yield parameters in the different structures. As the water was reduced from optimal level (100% ETc) to 80 or 60% ETc, there was a clear-cut decrease in fruit number, weight and the yield per plant. The interaction effect of the protected structure and irrigation level on fruit yield revealed that tomato fruit yield was distinctly higher at 100% ETc than at the deficit irrigation levels in all the protected structures, but no difference in yield was noticed between the two deficit levels. Further, at 80% ETc level of irrigation, there was no significant difference in yield between tomatoes grown in NVP or IPN in both years (Table 9; Figure 2). Overall, the lowest fruit yield was recorded in plants grown under SNH and exposed to the lowest ETc level (60%).

### 3.6. Water Productivity

**Figure 3:**
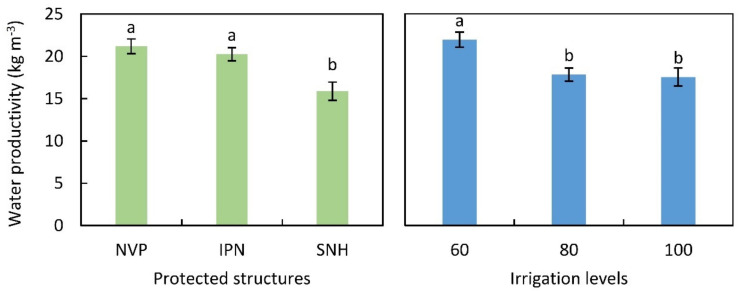
Water productivity (kg m^−3^) of tomato in different protected structures (**left**) and irrigation levels (**right**). Mean values followed by the same letter are not significantly different within each factor at *p* ≤ 0.05 according to DMRT. NVP, Naturally ventilated polyhouse; IPN, Insect-proof net house; and SNH, Shade net house. Irrigation levels 100, 80 and 60 correspond to 100%, 80% and 60% of ETc.

Water productivity (WP) was significantly influenced by protected structures and irrigation levels. The WP was slightly higher in 2020 than 2019; however, the trend observed was similar among the structures (data not presented). The two-year pooled data exhibited that the WP was recorded highest in NVP but was at par with IPN, and it was 33% and 28% higher than SNH, respectively (Figure 3). Regardless of the structures, plants exposed to deficit irrigation displayed higher WP as compared to the normal irrigation level (100% ETc).

## 4. Discussion

In the present investigation, there were clear-cut variations in different microclimatic variables among three protected cultivation structures (NVP, IPN and SNH) during the cropping period of the spring–summer tomato crop of 2019 and 2020. Almost similar trends in the measured microclimate variables (temperatures, RH and PAR) were observed during the period of investigation from January to May in both years (Table 1). The variations in microclimate parameters among the protected structures varied due to the different properties of the cladding materials of the structures, mainly influencing the passing of the solar radiation. This resulted in an alteration of light intensity and quality, and of other parameters such as temperature and RH. These changes can considerably influence the plants’ physiological processes, and subsequently plant growth and development inside the structures. The microclimate inside these low-cost protected cultivation structures can fluctuate, as they are often affected and dependent upon outside environments and locations [3,22]. Temperature is the most important determinant of plant growth and fruit set in tomatoes. The plants grown in NVP could have maintained better assimilation as it is directly linked with change in temperature [23]. The upper critical temperature limit in the tomato set is 35 °C; further, this affects pollen tube germination and fertility. However, high temperature with low RH may increase the vapor pressure deficit (VPD). This event was observed in net houses due to the porous nature of nets leading to escape of humidity. On the contrary, in NVP—due to poly-film—the RH was able to be retained inside the structure. This could lead to lower VPD inside the NVP in comparison to the net house; the plant canopy trait was significantly higher in this structure (Table 3 and Table 4). Our findings are consistent with those of Leyva et al. [24] who highlighted that a screen house with a plastic sheet reduced maximum temperature, increased RH and lowered VPD when a tomato crop was grown in the Spanish province of Granada during the spring and summer.

The superior performance of canopy traits, such as leaf DM (7% and 15%) and stem DM (28% and 31%) higher in NVP over IPN and SNH, respectively, was presumably due to better soil–plant–water relations that in turn related to efficient use of water. The differences in the vegetative traits among the structures was strongly associated with the effect of micro-climatic variables and light transmitted and modified by the cladding material [25]. In our previous work, the performance of greenhouse cucumbers, grown under low-tech structures during August–November, was superior in NVP; this was ascribed to better nutrient status as well as physiological processes (chlorophyll, F_v_/F_m_ and WP) of cucumber plants [26], favored by more congenial soil and air temperatures, humidity, and PAR availability in NVP than in net houses [3,26]. In the present study, the PAR availability of the tomato during the vegetative stage was optimum in NVP and INH and produced photo-assimilate more efficiently. The optimal microclimatic variables accounted for more biomass accumulation that led to better growth of the plants in NVP. Another important factor was that the poly-film cladded on NVP can diffuse the incoming radiation and penetrate deeper into the canopy of the plant to produce more photo-assimilates [27]. In the early growth period, the PAR levels were low in SNH. This might have elongated the cells and contributed to maximal leaf area at par with NVP. From most of the studies it was inferred that when the plant receives the optimum level of radiation with the congenial micro-climatic variables, it will produce the compact canopy that, in our research, was manifested by higher node number in NVP and INH housing, whereas the plants grown in the SNH had lesser node number and higher inter-nodal length [28]. Further, the stem thickness of the tomato plants in NVP was 4% and 15% higher over IPN and SNH due to greater vigor and biomass based on the pooled analysis. Overall, the primarily light with ambient temperature along with optimum humidity levels resulted in better canopy traits of the tomato plants grown in the NVP. It was very clear that, as the amount of irrigation water was reduced, it ultimately affected plant growth in all the protected environment structures. Our findings are in accordance with those stated by Khapte et al. [26] in cucumbers, Guilioni et al. [29] in peas, Kirnak et al. [30] in eggplants and Hassan et al. [31] in tomatoes.

The modulated micro-environment in the structures also altered the fruit’s physical attributes. The better growth of the plant in NVP could have produced more photo-assimilate and utilized it more efficiently, which is reflected in the higher fruit longitudinal (4 and 5%) and transverse diameter (2% and 6%) with maximum fruit firmness (6% and 8%) over IPN and SNH, respectively. These higher values for fruit physical parameters can be ascribed to the more efficient translocation of photo-assimilates in the plants grown in the NVP and the better source-sink movement, and it is also related to assimilate availability [32,33]. The plants in NVP could have better acquisition of water and nutrients due to a proper soil–plant–water balance and a favorable micro-environment, resulting in bigger fruits, as also observed in previous work on cucumbers [26]. In addition, the higher firmness of fruits from the NVP structure has more importance from post-harvest perspectives, such as transportation, marketability and storage of the produce. Therefore, the fruit’s physical attributes are more important in a tomato crop grown in arid and semi-arid regions. Our results also showed that, regardless of the structures, fruits from less irrigated plots had the highest fruit firmness values. This is totally in line with the results reported by Patanè and Cosentino [14] who, by studying the influence of water deficit on the productivity and the quality of processing tomatoes in a Mediterranean environment, found that fruits from non-irrigated plots revealed the highest firmness.

Microclimate variables such as air temperature, RH and PAR were relatively favorable in NVP, which resulted in a positive response not only in vegetative growth and fruit physical parameters but also in fruit quality parameters (Table 6). The fruit dry matter (DM), total soluble solids (TSS) and lycopene content in fruit were considerably higher in NVP. Temperature and radiation have a direct effect on the biosynthesis of lycopene development in tomato fruit [18]. Higher fruit lycopene in tomato fruits is corroborated with optimal temperature and PAR during the fruit-ripening stages. In addition to this, the effect of diffused radiation in NVP produces an advantage in the biosynthesis of lycopene and fruit color (Table 6 and Table 7). Nangare et al. [34] observed an increasing trend for fruit quality parameters such as fruit firmness, TSS and color with increased water deficit level in tomato. Further, it was observed that the fruit from SNH had higher total carotenoids that can be correlated with the higher L* and b* values, which is an indication of black-white and yellow-blue coordinates, respectively. Besides this, the level of carotenoid accumulation in fruits is affected by temperature and light intensity. When temperatures rise above 32 °C, lycopene accumulation is inhibited and converted into carotene [35]. Overall, fruit quality from NVP was exceptionally better with uniform red color fruit with higher firmness than those from other structures. Our results also showed that well-watered treatment (100% ETc) significantly enhanced lycopene concentration in tomato fruits. These results are in line with those reported by Helyes et al. [36] who, by investigating the influence of irrigation management on processing tomato yield and antioxidant compounds, found that fruits from regularly irrigated plots had the highest lycopene concentration.

The protected environment structures modulated the micro-environment, which ultimately influenced the yield parameters. Higher fruit number and fruit weight were due to profuse flowering and fruit setting in the NVP and IPN due to the optimal range of micro-climatic variables during the flowering stage, in the months from February to April. In tomatoes, fruit setting is directly correlated to the ambient temperature, because poor fruit set at high temperatures affect pollen viability and germination due to reduced stigma receptivity [37]. Even though temperature during the flowering stage was up to 36 °C, there was better RH in the NVP and IPN; therefore, the stigma receptivity might be better, which could lead to better fruit set and an increase in fruit number per plant. Continuous high temperature with low RH can impair the reproductive rate of fruit development in SNH [30,38]. Maximum fruits per plant with higher mean fruit weight contributed to the total fruit yield, which was at par in NVP and IPN and apparently higher as compared to SNH. Under shading conditions with optimum temperature, plants can use water and nutrients more efficiently; this in turn can enhance the fruit yield in tomatoes [39]. The physiological parameters were also influenced by the altered microclimate. Plants inside the NVP had better leaf RWC, higher PSII efficiency and significantly lower water potential because those grown in NVP were able to acquire water most efficiently and maintain better soil–plant water balance. The ability of plants in NVP to maintain superior physiological functioning was further substantiated by beneficial diffuse radiation that penetrates deep into the canopy, whereas direct radiation is harmful and may negatively impair photosynthetic activity of plants in net houses [40]. Further, the higher yield in NVP is also ascribed to the cladding of plastic sheeting on the NVP, which helps accumulate CO_2_ for photosynthesis, whereas in net houses CO_2_ escapes [41,42]. Likewise, the altered microclimate in a protected structure (i.e., NVP) and its positive influence on plant physiological processes and consequently on growth and yield was also reported in cucumbers [26].

Water productivity is particularly significant in arid and semi-arid areas where water is scarce. In both years, the highest water productivity was noticed in NVP with no significant deviation from IPN. The favorable microclimate in these structures, together with high plant–water balance, might have induced a superior plant physiological performance in tomato plants. Photo-assimilates could also be used effectively in these structures to increase fruit output while conserving water. Since in our previous work on greenhouse cucumbers, a significant variation in water productivity was evident between NVP (higher) and IPN (lower) [26], the performance of tomatoes needs also to be tested in other seasons in order to have more comprehensive information on protected cultivation in Indian (semi) arid regions.

## 5. Conclusions

In arid climates, the naturally ventilated polyhouse (NVP) and insect-proof net house (IPN) were found to be better than the shade net house (SNH) for spring–summer tomato cultivation. Plants cultivated in NVP had a more favourable microclimate followed by those in IPN. This could have favored satisfactory plant growth, fruit yield parameters as well as fruit physico-chemical qualities during spring–summer. Furthermore, plant physiological state was better in NVP due to higher leaf RWC, better PSII efficiency and lower water potential, indicating that the soil–plant–water balance was optimal and that the plants were not stressed. As for yield, NVP performed better in terms of ETc (100%) and for water productivity, and both NVP and IPN performed similarly; however, fruits from NVP exhibited better quality attributes in addition to yield. Overall, NVP is an appropriate protected cultivation structure with a 100% ETc irrigation level in order to improve fruit quality and increase production with efficient water management for spring–summer tomato crops cultivated in resource-scarce arid regions.

## Figures and Tables

**Table 1:** Weather data (air temperature, relative humidity and PAR) of tomato growing period (spring–summer 2019 and 2020).

Year/Months	Max. Temperature (°C)			Min. Temperature (°C)			Mean RH (%)			PAR (µmol.m^−2^s^−1^)		
	NVP	INH	SNH	NVP	INH	SNH	NVP	INH	SNH	NVP	INH	SNH
2019												
January	25.14	21.43	21.86	12.16	11.26	11.48	53.63	49.82	47.81	561.17	556.08	314.54
February	28.13	26.67	27.00	13.34	12.01	12.87	47.37	44.98	42.67	742.63	720.38	414.00
March	31.68	31.08	31.64	17.12	17.96	18.37	38.06	35.42	32.91	716.80	689.78	463.78
April	36.82	37.00	38.14	24.31	25.68	25.67	29.92	26.57	25.36	353.51	421.74	508.17
May	39.00	39.18	40.32	26.81	28.05	28.68	36.28	34.61	32.41	317.25	477.31	563.69
2020												
January	24.15	22.22	21.96	13.02	11.98	11.74	58.82	55.91	54.89	538.83	517.88	292.03
February	28.76	28.10	28.05	13.11	12.76	13.05	49.65	45.03	44.87	622.91	705.00	391.03
March	27.43	27.43	27.36	16.46	17.08	18.67	42.81	40.18	39.24	718.83	804.83	480.00
April	36.33	35.00	35.00	23.89	25.27	25.89	36.01	34.23	32.15	336.69	520.42	560.35
May	38.50	38.18	39.82	25.92	28.12	28.23	34.64	31.69	30.54	347.25	537.31	573.69

NVP, Naturally ventilated polyhouse; IPN, Insect-proof net house; and SNH, Shade net house.

**Table 2:** Total amount of applied water in years 2019 and 2020 during tomato growth period in protected structures.

Irrigation Levels	Total Water Applied via Drip System (cm)	
	2019	2020
100% ETc	67.95	64.07
80% ETc	54.36	51.26
60% ETc	40.77	38.44

Note: The common irrigation 6.00 cm was applied from transplanting to 21 days for proper establishment of seedlings during 2019 and 2020.

**Table 3:** Effect of protected structures and irrigation levels on canopy traits of tomato (plant^−1^).

Treatments	Stem Dry Matter (g)			Leaf Area (m^2^)		
	2019	2020	Pooled	2019	2020	Pooled
Structures (S)						
NVP	41.76 a	46.63 a	44.20 a	0.90 b	1.06 a	0.98 a
IPN	33.21 b	35.53 b	34.37 b	0.85 c	0.91 c	0.88 b
SNH	32.44 b	35.05 b	33.74 b	0.95 a	0.99 ab	0.97 a
Irrigation (I)						
100% ETc	40.58 a	43.79 a	42.18 a	0.96 a	1.05 a	1.01 a
80% ETc	35.01 b	37.91 b	36.46 b	089 b	0.99 ab	0.94 b
60% ETc	31.83 c	35.51 b	33.67 c	0.85 b	0.93 b	0.89 c
S	***	***	***	**	**	**
I	***	***	***	***	*	**
S × I	*	*	NS	NS	NS	NS

Mean values of three replicates followed by the same letter for each factor within each column are not significantly different according to DMRT *p* ≤ 0.05. NS, Non-significant; Significance *, **, *** at *p* ≤ 0.05, 0.01 and 0.001, respectively. NVP, Naturally ventilated polyhouse; IPN, Insect-proof net house; and SNH, Shade net house.

**Table 4:** Effect of protected structures and irrigation levels on growth parameters of tomato (plant^−1^).

Treatments	Plant Height (cm)			Node Number			Stem Girth (mm)		
	2019	2020	Pooled	2019	2020	Pooled	2019	2020	Pooled
Structures (S)									
NVP	230.30 a	255.95 a	243.13 a	31.83 a	35.71 a	33.77 a	12.55 a	10.60 a	11.57 a
IPN	230.86 a	234.45 b	235.25 b	31.13 a	34.34 ab	32.74 a	11.96 b	10.35 b	11.16 b
SNH	227.05 a	249.38 ab	240.12 ab	28.86 b	33.93 c	31.40 b	10.90 c	9.24 c	10.07 c
Irrigation (I)									
100% ETc	238.94 a	256.66 a	247.80 a	31.52 a	36.50 a	34.01 a	12.25 a	10.62 a	11.43 a
80% ETc	234.36 b	252.86 a	243.61 a	30.95 a	34.12 b	32.53 b	11.67 b	9.89 b	10.78 b
60% ETc	214.91 c	239.27 b	227.09 b	29.36 a	33.36 b	31.36 c	11.50 b	9.67 b	10.59 b
S	NS	*	*	*	*	**	***	***	***
I	***	***	***	NS	***	***	*	***	***
S × I	NS	NS	NS	NS	NS	NS	NS	NS	NS

Mean values of three replicates followed by the same letter for each factor within each column are not significantly different according to DMRT *p* ≤ 0.05. NS, Non-significant; Significance *, **, *** at *p* ≤ 0.05, 0.01 and 0.001, respectively. NVP, Naturally ventilated polyhouse; IPN, Insect-proof net house; and SNH, Shade net house.

**Table 5:** Effect of protected structures and irrigation levels on fruit physical parameters of tomato.

Treatments	Longitudinal Diameter (cm)			Transverse Diameter (cm)			Fruit Firmness (kg cm^−2^)		
	2019	2020	Pooled	2019	2020	Pooled	2019	2020	Pooled
Structures (S)									
NVP	4.90 a	4.36 a	4.63 a	6.08 a	5.85 a	5.96 a	3.18 a	3.23 a	3.20 a
IPN	4.79 b	4.23 b	4.51 b	5.97 b	5.72 a	5.85 b	2.95 b	3.09 a	3.02 b
SNH	4.73 b	4.06 c	4.39 c	5.76 c	5.45 b	5.60 c	3.05 ab	2.86 b	2.95 b
Irrigation (I)									
100% ETc	4.92 a	4.45 a	4.69 a	6.13 a	5.90 a	6.02 a	2.94 b	2.93 b	2.94 b
80% ETc	4.78 b	4.15 b	4.47 b	5.91 b	5.65 b	5.78 b	3.01 b	3.09 ab	3.04 b
60% ETc	4.71 b	4.04 c	4.37 c	5.77 c	5.46 c	5.62 c	3.24 a	3.15 a	3.19 a
S	**	***	***	***	***	***	*	***	***
I	***	***	***	***	***	***	***	*	***
S × I	NS	NS	NS	NS	NS	NS	NS	NS	NS

Mean values of three replicates followed by the same letter for each factor within each column are not significantly different according to DMRT *p* ≤ 0.05. NS, Non-significant; Significance *, **, *** at *p* ≤ 0.05, 0.01 and 0.001, respectively. NVP, Naturally ventilated polyhouse; IPN, Insect-proof net house; and SNH, Shade net house.

**Table 6:** Effect of structures and irrigation levels on fruit quality parameters of tomato.

Treatments	Fruit DM (%)			TSS (°Brix)			Total Carotenoids (mg 100 g^−1^ FW)			Lycopene (mg 100 g^−1^ FW)		
	2019	2020	Pooled	2019	2020	Pooled	2019	2020	Pooled	2019	2020	Pooled
Structures (S)												
NVP	6.02 a	4.89 a	5.93 a	5.01 a	4.89 a	4.73 a	4.84 b	4.14 c	4.49 c	2.64 a	2.49 a	2.57 a
IPN	6.01 a	4.11 b	5.89 a	4.32 b	4.11 b	4.34 b	7.11 a	5.70 b	6.41 b	2.26 ab	2.08 b	2.17 b
SNH	5.90 a	4.00 b	5.66 b	4.33 b	4.00 b	4.22 b	7.01 a	7.15 a	7.08 a	1.94 b	1.86 b	1.90 c
Irrigation (I)												
100% ETc	5.79 b	4.21 a	5.64 b	4.11 b	4.22 a	4.23 b	5.68 b	5.12 c	5.40 b	2.38 a	2.28 a	2.33 a
80% ETc	5.94 ab	4.33 a	5.77 b	4.56 ab	4.31 a	4.41 b	6.45 a	5.73 b	6.09 a	2.26 a	2.16 ab	2.21 ab
60% ETc	6.20 a	4.45 a	6.08 a	5.00 a	4.43 a	4.65 a	6.83 a	6.14 a	6.48 a	2.20 a	1.99 b	2.10 c
S	NS	***	*	*	***	***	***	***	***	**	***	***
I	*	NS	**	**	NS	**	**	***	***	NS	*	*
S × I	NS	NS	NS	NS	NS	NS	NS	*	*	NS	NS	NS

Mean values of three replicates followed by the same letter for each factor within each column are not significantly different according to DMRT *p* ≤ 0.05. NS, Non-significant; Significance *, **, *** at *p* ≤ 0.05, 0.01 and 0.001, respectively. NVP, Naturally ventilated polyhouse; IPN, Insect-proof net house; and SNH, Shade net house. DM and TSS, dry matter and total soluble solids, respectively.

**Table 7:** Effect of structures and irrigation levels on fruit color of tomato fruit (in 2020).

Treatments	L* (Hue)	a* (% of Red)	b* (% of Yellow)
Structures (S)			
NVP	35.00 b	16.92 a	16.14
IPN	35.07 b	16.11 b	16.23
SNH	35.70 a	14.85 c	16.70
Irrigation (I)			
100% ETc	35.64 a	15.15 c	15.99
80% ETc	35.28 ab	16.03 b	16.40
60% ETc	34.85 b	16.69 a	16.68
S	**	***	NS
I	**	***	NS
S × I	*	NS	NS

Mean values of three replicates followed by the same letter for each factor within each column are not significantly different according to DMRT *p* ≤ 0.05. NS, Non-significant; Significance *, **, *** at *p* ≤ 0.05, 0.01 and 0.001, respectively. NVP, Naturally ventilated polyhouse; IPN, Insect-proof net house; and SNH, Shade net house.3.5. Plant Physiological Parameters

**Table 8:** Effect of protected structures and irrigation levels on physiological parameters of tomato.

Treatments	RWC (%)			Total Chlorophyll (µg mL^−1^)			Chlorophyll Fluorescence (F_v_/F_m_)			LWP (-bar)		
	2019	2020	Pooled	2019	2020	Pooled	2019	2020	Pooled	2019	2020	Pooled
Structures (S)												
NVP	71.30 ab	79.48 a	75.39 a	15.99 b	12.95 b	14.47 b	0.83 a	0.82 a	0.82 a	8.75 c	10.89 a	9.81 c
IPN	68.49 b	74.52 b	71.50 b	17.53 a	14.37 a	15.95 a	0.79 b	0.80 b	0.80 b	9.83 b	11.12 a	10.47 b
SNH	74.18 a	69.96 c	72.07 b	17.76 a	14.33 a	16.05 a	0.76 c	0.80 b	0.78 c	10.50 a	11.22 a	10.86 a
Irrigation (I)												
100% ETc	73.75 a	76.70 a	75.22 a	16.26 b	12.148 c	14.37 c	0.80 a	0.82 a	0.81 a	8.58 c	9.67 c	9.12 c
80% ETc	70.88 a	74.04 b	72.46 b	17.11 ab	13.87 b	15.49 b	0.80 a	0.81 a	0.80 a	9.83 b	10.67 b	10.25 b
60% ETc	69.34 a	73.22 c	71.28 b	17.92 a	15.31 a	16.61 a	0.79 a	0.78 b	0.79 b	10.66 a	12.89 a	11.77 a
S	*	***	**	**	*	***	***	*	***	***	NS	***
I	NS	*	**	**	***	***	NS	***	**	***	***	***
S × I	NS	NS	NS	NS	NS	*	*	*	*	*	NS	NS

Mean values of three replicates followed by the same letter for each factor within each column are not significantly different according to DMRT *p* ≤ 0.05. NS, Non-significant; Significance *, **, *** at *p* ≤ 0.05, 0.01 and 0.001, respectively. NVP, Naturally ventilated polyhouse; IPN, Insect-proof net house; and SNH, Shade net house. RWC and LWP, relative water content and leaf water potential, respectively.

**Table 9:** Effect of protected structures and irrigation levels on yield parameters of tomato.

Treatments	Fruit Number (No. Plant^−1^)			Mean Fruit Weight (g Fruit^−1^)			Total Fruit Yield (kg Plant^−1^)		
	2019	2020	Pooled	2019	2020	Pooled	2019	2020	Pooled
Structures (S)									
NVP	54.45 a	56.38 a	55.42 a	78.71 a	80.19 a	79.45 a	3.22 a	3.41 a	3.31 a
IPN	51.66 a	58.22 a	54.94 a	73.40 b	75.51 b	74.46 b	3.05 a	3.23 b	3.14 a
SNH	39.25 b	42.88 b	41.06 b	73.65 b	74.50 b	74.07 b	2.12 b	2.33 c	2.23 b
Irrigation (I)									
100% ETc	52.58 a	54.66 a	53.62 a	82.59 a	83.14 a	82.86 a	3.32 a	3.47 a	3.39 a
80% ETc	47.52 b	51.38 b	49.45 b	73.62 b	75.68 b	74.65 b	2.63 b	2.87 b	2.75 b
60% ETc	45.26 b	51.44 b	48.35 b	69.55 c	71.38 c	70.47 c	2.44 b	2.63 c	2.54 c
S	***	***	***	**	***	***	***	***	***
I	**	**	***	***	***	***	***	***	***
S × I	NS	NS	**	NS	NS	NS	NS	***	**

Mean values of three replicates followed by the same letter for each factor within each column are not significantly different according to DMRT *p* ≤ 0.05. NS, Non-significant; Significance **, *** at *p* ≤ 0.01 and 0.001, respectively. NVP, Naturally ventilated polyhouse; IPN, Insect-proof net house; and SNH, Shade net house.

## Data Availability

The datasets generated for this study are available on request to the corresponding authors.
